# Review of clinical and imaging findings in autoimmune glial fibrillary acidic protein astrocytopathy to aid in early diagnosis

**DOI:** 10.3389/fimmu.2024.1466847

**Published:** 2024-12-10

**Authors:** Xiaomeng Li, Jiacun Li, Han Xu, Xiaohui Liu, Meilin Li, Jingzhen He, Jianjun Xiu

**Affiliations:** ^1^ Department of Radiology, Shandong Provincial Hospital Affiliated to Shandong First Medical University, Jinan, Shandong, China; ^2^ Department of Radiology, People’s Hospital of RiZhao, RiZhao, Shandong, China; ^3^ Department of Radiology, Jinan Third People’s Hospital, Jinan, Shandong, China; ^4^ Department of Neurology, Shandong Provincial Hospital Affiliated to Shandong First Medical University, Jinan, Shandong, China; ^5^ Department of Radiology, The First Affiliated Hospital of Shandong First Medical University & Shandong Provincial Qianfoshan Hospital, Jinan, Shandong, China; ^6^ Department of Radiology, Qilu Hospital of Shandong University, Jinan, Shandong, China

**Keywords:** autoimmune glial fibrillary acidic protein astrocytopathy, meningeal enhancement, brain FLAIR gadolinium enhancement, spinal cord longitudinal T2 hyperintensity, overlapping antibodies

## Abstract

**Objective:**

Autoimmune glial fibrillary acidic protein astrocytopathy (GFAP-A) is a novel steroid sensitive autoimmune disease, without a diagnostic consensus. The purpose of this study was to improve early GFAP-A diagnosis by increasing awareness of key clinical characteristics and imaging manifestations.

**Methods:**

Medical records of 13 patients with anti-GFAP antibodies in serum or cerebrospinal fluid (CSF) were reviewed for cross-sectional and longitudinal analysis of clinical and magnetic resonance imaging (MRI) findings.

**Results:**

The predominant GFAP-A clinical manifestations are limb weakness/numbness and fever. GFAP-A has a propensity in the early stage for meningeal and leptomeningeal lesions on the brainstem surface, with a typical pattern of periventricular linear radial and leptomeningeal enhancement. The clinical manifestations and leptomeningeal enhancement were rapidly alleviated after treatment with high doses of corticosteroids or/and intravenous immunoglobulin, although, there are patients who may present with increased brain parenchymal lesions. On 3T MRI, the spinal cord demonstrated extensive longitudinal T2-weighted hyper-intensity, central distribution, and gray matter involvement. Optic nerve involvement in some patients was also noted with optic nerve swelling and abnormal enhancement. In addition to the classic reversible splenium of corpus callosum syndrome (type I), this study found the much rarer type II with diffusion restriction on DWI (Diffusion Weighted Imaging) in the corpus callosum. Positive anti-GFAP antibodies in serum or cerebrospinal fluid (CSF) are important for GFAP-A diagnosis with overlapping antibodies commonly noted. This study found anti-GM3 antibodies, a rare finding also previously reported.

**Conclusion:**

This study correlates GFAP-A clinical and imaging features, noting a “delay” phenomenon between clinical manifestations, treatment response, and radiographic MRI findings. MRI T2-FLAIR brainstem hyperintensity and T2-FLAIR gadolinium enhanced images, and subtraction techniques were valuable for early lesion detection and accurate diagnosis.

## 1 Introduction

Autoimmune glial fibrillary acidic protein astrocytopathy (GFAP-A) is a newly identified steroid hormone-sensitive meningoencephalomyelitis with specific antibody (GFAP-IgG), which was first reported by Fang et al. (1) in 2016. Glial fibrillary acidic protein (GFAP) is an intermediate filament protein of mature astrocytes. It is involved in the formation of cytoskeletal structures and cytoskeletal functions, such as cell movement and migration, proliferation, and the regulation of synaptic plasticity (2). Anti-GFAP antibody is the biomarker for the autoimmune response in this disease, it is CD8+ T cells that accelerate inflammatory central nervous system (CNS) autoimmunity (3, 4). Currently, the pathogenesis of Autoimmune GFAP astrocytopathy is unknown, with researchers hypothesizing the pathogenesis is due to involvement of interactions of T and B lymphocytes and immune inflammatory components (5). This autoimmune disease of the nervous system can present as various combinations of encephalitis, meningoencephalitis, myelitis, and optic neuritis. The main clinical manifestations are headache, fever, nausea, vomiting, and disturbance of consciousness (6). Although the clinical features and laboratory tests for this disease are described in numerous domestic and international studies, there are no clear international guidelines for its clinical diagnosis and treatment. Moreover, the disease heterogeneity and overlapping antibodies pose diagnostic challenges for clinicians, especially in early phases of the disease. The disease has relatively characteristic imaging findings with a radial linear enhancement pattern around the ventricles (1), with frequently observed leptomeningeal enhancement and with bilateral thalami and basal ganglia involvement as the most commonly affected sites (6). The aims of this study were to investigate the clinical characteristics and imaging manifestations of GFAP-A to enhance early diagnosis and improve understanding of the disease.

## 2 Subjects and methods

### 2.1 Subjects

A total of 13 patients with anti-GFAP antibodies in serum or cerebrospinal fluid (CSF) were enrolled from Shandong Provincial Hospital, Qianfoshan Hospital of Shandong Province, and Qilu Hospital of Shandong University. Inclusion criteria consisted of clinical symptoms of meningitis, encephalitis, or encephalomyelitis, and the presence of anti-GFAP antibodies in CSF or serum. Exclusion criteria were patients with positive anti-GFAP antibody in serum or CSF caused by craniocerebral or spinal cord trauma or tumor.

### 2.2 Laboratory and imaging examination

All patients underwent cerebrospinal fluid examination at least once. CSF cell count, protein content, glucose quantification, and chloride level were recorded for analysis. A cell-based assay (CBA) was used to detect anti-GFAP antibodies in serum or CSF of patients. Central nervous demyelinating antibodies (anti-AQP4 antibody, anti-MOG antibody, and anti-MBP antibody) were assessed in 10 of patients, 7 patients had autoimmune encephalitis antibodies (anti-NMADR, anti-AMPA1, anti-AMPA2, anti-LGI1, anti-CASPR2, and anti-GABAB), ganglioside antibodies, and paraneoplastic antibodies (anti-Hu, anti-Y, anti-CV, anti-PNMA2, anti-Amphiphysin, anti-Ma1, anti-SOX1, anti-Tr, Zic4, anti-GAD65 anti-, PKCγ, anti-Recoveri, and anti-Titin). CSF and sera of patients were tested for pathogens such as cytomegalovirus, Epstein-Barr virus, Coxsackie virus, measles virus, herpes simplex virus, herpes zoster virus, rubella virus, echovirus, human parvovirus b-19, influenza virus, and *Mycoplasma pneumoniae* to exclude central nervous system infection. Chest computed tomography (CT)was conducted for all patients, while abdominal CT and abdominal ultrasound were performed in 10 and 4 patients respectively to exclude neoplastic disease. The demographic characteristics, clinical presentation, hematological parameters, biochemical profiles, radiographic findings, and therapeutic regimens of the subjects were retrospectively analyzed. All subjects underwent at least one cranial and/or spinal MRI with contrast prior to treatment. Gadolinium (gadobenate dimeglumine Injection, 0.1 mmol/Kg) contrast-enhanced MRI was performed in 12 patients. Three patients received gadolinium for imaging with T2-FLAIR gadolinium enhanced sequences on a 3.0T uMR 790 scanner. (United-Imaging, Shanghai, China) The detailed parameters of MRI acquisition are summarized in **Supplementary Table S1**. All subjects were assessed and first examined by MR within 2 weeks of admission for acute episodes. The modified Rankin Scale (mRS) was employed to assess the severity of the disease and therapeutic effect at admission and discharge.

## 3 Results

### 3.1 Demographic data and clinical manifestations

Demographic and clinical characteristics of the patients are summarized in **Table 1**. Eight males and four females were enrolled in the study. Age of disease onset ranged from 10 years to 71 years (median 38 years). The clinical manifestations included limb weakness/numbness (11/13), fever (10/13), nausea/vomiting (8/13), dizziness/headache (7/13), bowel dysfunction (7/13), tremor (4/13), memory loss (4/13), seizures (3/13), visual changes (3/13), weight loss (2/13), visual hallucinations, and auditory hallucinations (1/13). Prodromal symptoms were observed in 12 of 13 patients, all of whom experienced an unprovoked fever onset 8 days to 5 months before the acute phase, with a peak body temperature of 40°C. In this cohort, two patients had confirmed history of recurrence, one patient with one and an additional patient with three recurrences, three patients were admitted to intensive care, and one patient had a prior history of psoriasis (#2).

**Table 1:** Clinical features, diagnosis, antibodies, treatment strategies and relapse in patients positive for GFAP-IgG.

No./sex	Age	Prodromal symptoms	Initial diagnosis	Anti-GFAP antibody and titer		Other Antibodies	Treatment	Modified mRS Score Admission/Discharge	ICU	Relapse
				Cerebrospinal fluid	Serum					
1/Female	28	Fever	Intracranial infection, viral encephalitis	/	1:32	Serum anti-GM3 antibody positive	IVMP+IVIG	5,4	Yes	0
2/Male	21	Fever	Reversible splenial lesion syndrome, viral meningoencephalitis	1:1+	Negative	Cerebrospinal fluid anti- AQP4 antibody positive	IVMP+IVIG	3,2	No	0
3/Male	46	Fever	Central nervous system infection, autoimmune encephalitis	1:3.2 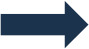 1:1	Negative	Serum anti-MOG antibody positive	IVMP	5,3	Yes	0
4/Female	46	Fever	Transverse myelitis,multiple sclerosis	/	1:32	Negative	IVMP	3,1	No	0
5/Male	10	Fever	Meningitis	1:10 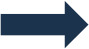 Negative	Negative	Negative	IVMP+IVIG	2,1	Yes	0
6/Male	44	Fever	Undiagnosed fever, intracranial infection	1:10++ 1:10+ 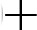	1:320 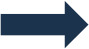 1:10+	Negative	IVMP+IVIG	5,2	No	0
7/Male	71	Fever	Undiagnosed limb weakness, prostatic hyperplasia, urinary tract infection	1:1++	Negative	Serum anti-NMDAR antibody positive	IVMP	3,2	No	0
8/Male	57	Fever	Urinary tract infection, enteritis	1:100 1:32 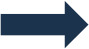	1:100 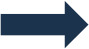 1:10	Negative	IVMP	2,1	No	0
9/Female	32	Fever	Viral meningitis	1:10	Negative	Negative	IVMP	3,2	No	Three times
10/Female	22	Fever	Intracranial infection	1:10+	1:100+	Negative	IVMP+IVIG	4,2	No	0
11/Male	29	Fever	Ataxia reason pending investigation	1:1+	Negative	Negative	IVMP	4,2	No	One time
12/Male	61	No	Spinal cord lesions, Alzheimer's disease	1:32	Negative	Negative	IVMP	5,3	No	0
13/Male	33	Fever	Intracranial infection	1:100	Negative	Serum anti-NMDAR antibody positive	IVMP+IVIG	5,4	No	0

### 3.2 Laboratory examination

#### 3.2.1 Anti-GFAP antibody and additional antibodies

All 13 patients were positive for anti-GFAP antibodies in serum or CSF, including two patients with only serum GFAP antibodies. Five patients had overlapping antibodies in that one patient had positive MOG antibodies in CSF, one patient had AQP4 antibodies in CSF, one patient had anti-GM3 antibodies in serum, and two patients had NMDAR specific antibodies in serum (**Table 1**).

#### 3.2.2 Characteristics of CSF

All patients underwent lumbar puncture with routine and biochemical analysis of CSF, with indications and findings recorded. CSF appearance was colorless and clear/transparent except for one patient (#8) specimen that had a red and turgid appearance. There were 12 patients (12/13) with increased CSF protein content (median: 1.335 g/L, range: 0.66-4.99 g/L, normal: 0.15-0.45 g/L). These 12 patients (12/13) had increased CSF white blood cell (WBC) counts (median: 126.5×10*6/L, range: 12.0-717.0×10*6/L, normal: 0-8×10*6/L). One patient (#9) had a normal WBC count upon admission. CSF chloride was decreased in 10 patients (10/13) and within the normal range for three patients (median: 115.8 mmol/L, range: 106-118.8 mmol/L, normal: 120-135 mmol/L). Three patients (4/13) had decreased CSF glucose levels (minimum 2.275 mmol/L, normal: 2.5-4.5 mmol/L). Four patients were positive for oligoclonal band type 2 in their CSF. Four patients had undergone next generation sequencing (NGS) of their CSF, which revealed that one patient was infected with the Epstein-Barr virus, one patient was infected with the *Epstein-Barr* virus and *Aspergillus flavus*, one patient was infected with *Bronchial gordonia*, and one patient was infected with *Pseudomonas aeruginosa*, *Bacillus palpis intermedia*, and *Staphylococcus* (**Table 2**).

**Table 2:** Serum and CSF findings of 13 patients with autoimmune GFAP astrocytopathy.

	serum						Cerebrospinal fluid							
No./sex	Na mmol/L	K mmol/L	Cl mmol/L	Other immunologic rheumatic antibodies	Other Pathogens	Tumor markers	Color	Transparency	Protein Content g/L	Glucose mmol/L	Chloride chloride mmol/L	Cell count 10*6/L	Other Pathogens	Oligoclonal band
1/Female	135.6	3.2	106.4	Negative	Influenza virus;Bunya virus;	/	Color	Clarity	1.15	4.43	116.7	156	/	/
2/Male	135.8	3.79	102	Negative	/	/	Color	Clarity	1.54	2.83	118.1	298	Aflatus/Aspergillus oryzae; Epstein-Barr virus	/
3/Male	127.2	4.42	91.6	/	Epstein-Barr virus; Mycoplasma pneumoniae.	/	Color	Clarity	1.55	3.04	106	266	/	/
4/Female	133	3.66	100	Negative	Negative	Negative	Color	Clarity	1.34	2.55	114.9	60	/	/
5/Male	133.4	3.59	103	/	Epstein-Barr virus; Mycoplasma pneumoniae.	Neuron-Specific enolase	Color	Clarity	0.87	2.14	118	137	/	/
6/Male	128	3.39	98	Negative	Rubella virus; Cytomegalovirus. Herpes simplex virus; Parvovirus B19.	Negative	Color	Turbidity	4.99	2.66	108.5	717	Pseudomonas aeruginosa, Pale Bacillus intermedia, Staphylococcus	Positive
7/Male	134.2	3.29	95.8	Negative	Rubella virus; Cytomegalovirus. Herpes simplex virus	Carcinoembryonic antigen; Carbohydrate antigen 125; Ferritin; Neuron-Specific enolase	Color	Clarity	1.28	2.88	117.9	64	/	Positive
8/Male	128	4.09	90.6	Negative	Epstein-Barr virus	Neuron-Specific enolase	Color	Clarity	1.94	2.6	118.8	116	Epstein-Barr virus	/
9/Female	127	3.95	92.4	Negative	Cytomegalovirus; Herpes simplex virus;Epstein-Barr virus.	Carbohydrate antigen-124	Color	Clarity	1	2.41	111	5	Gordonia bronchi	Positive
10/Female	131	4.19	101	/	Cytomegalovirus; Herpes simplex virus; Rubella virus	Neuron-Specific enolase; Carbohydrate antigen-125	Color	Clarity	0.66	2.48	124	12	/	/
11/Male	137	4.21	101	/	Cytomegalovirus; Herpes simplex virus; Epstein-Barr virus.	Neuron-Specific enolase; Non-small cell lung cancer associated antigen	Color	Clarity	0.34	5.29	127	142	/	Positive
12/Male	140	3.82	105	/	/	Negative	Color	Clarity	1.33	3.41	124	40	/	Negative
13/Male	125.7	4.03	94.3	Negative	Cytomegalovirus; Herpes simplex virus.	Neuron-Specific enolase	Color	Clarity	1.93	1.56	113.3	115	Negative	/

#### 3.2.3 Serological examination

The majority of patients n=11 had hyponatremia, n=6 had hypochloremia, and n=3 had hypokalemia. Serum pathogens were detected in 11 patients, including cytomegalovirus (6/11), herpes simplex virus (6/11), and *Epstein-Barr virus* (5/11). Rubella virus (2/11), *Mycoplasma pneumoniae* (2/11), influenza virus (1/11), bunyavirus (1/11), *parvovirus B19* (1/11), *Staphylococcus epidermidis* (1/11), and *Enterococcus cecum* (1/11) were found in a minority of patients. Only one patient had no bacterial or viral infections. Ten patients were examined for tumor markers, of which six patients (6/10) had abnormal increases in Neuron-Specific enolase, but no tumors were detected. Eight patients tested negative for other non-neurological autoimmune antibodies, including anti-nuclear, anti-neutrophil cytoplasmic, anti-phospholipid, rheumatoid factor, and anti-dsDNA antibodies (**Table 2**).

### 3.3 Imaging findings

**Figure 1:**
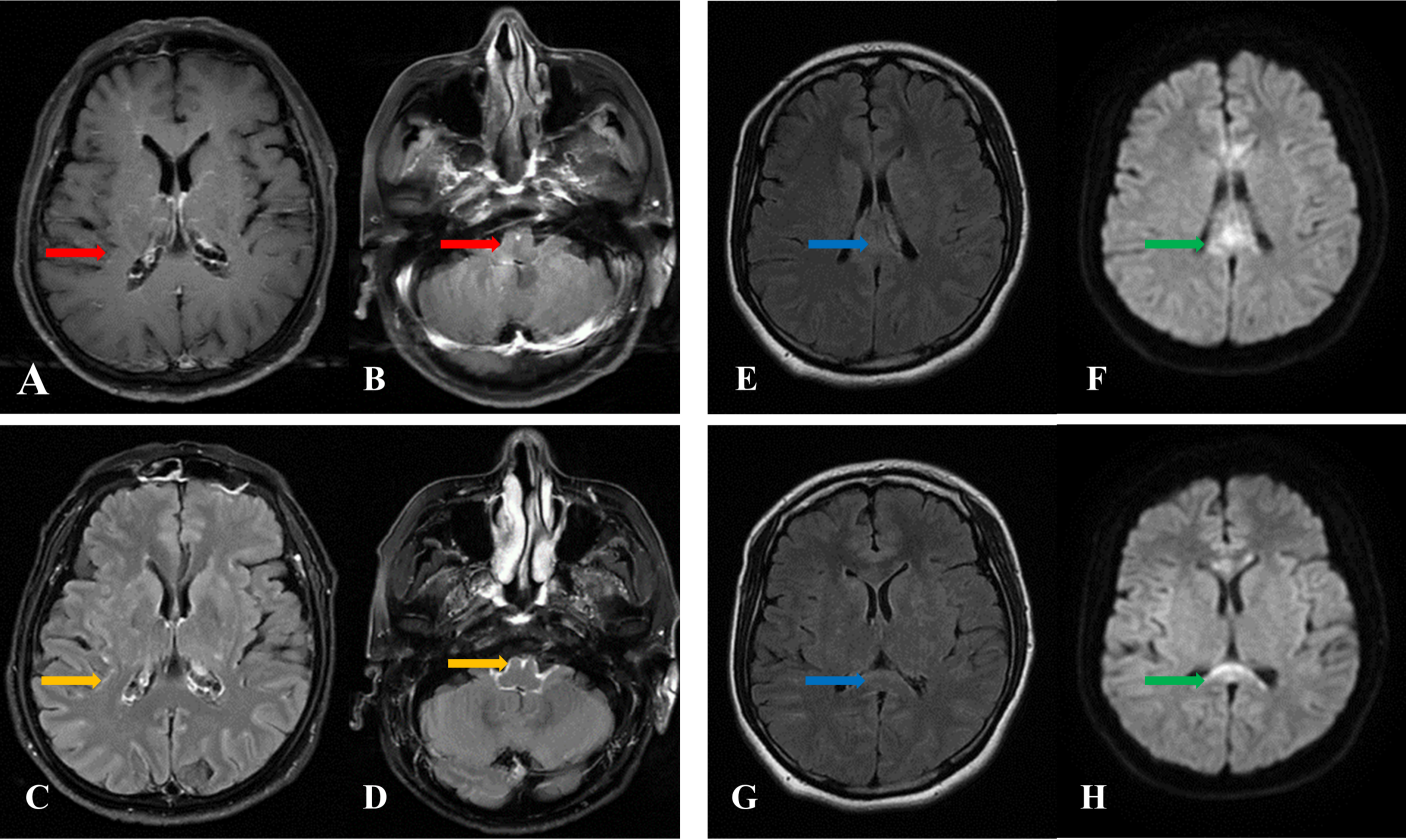
**(A, B)** Axial T1 gadolinium contrast enhanced MRI of the brain, patient #8 **(C, D)** Axial T2 weighted fluid attention (T2-FLAIR) post contrast, demonstrating linear intraparenchymal and leptomeningeal enhancement. **(E, G)** Axial T2-FLAIR, and **(F, H)** diffusion weighted imaging (DWI), with patchy T2-FLAIR and DWI hyperintensity in the corpus callosum, patient #1.

**Figure 2:**
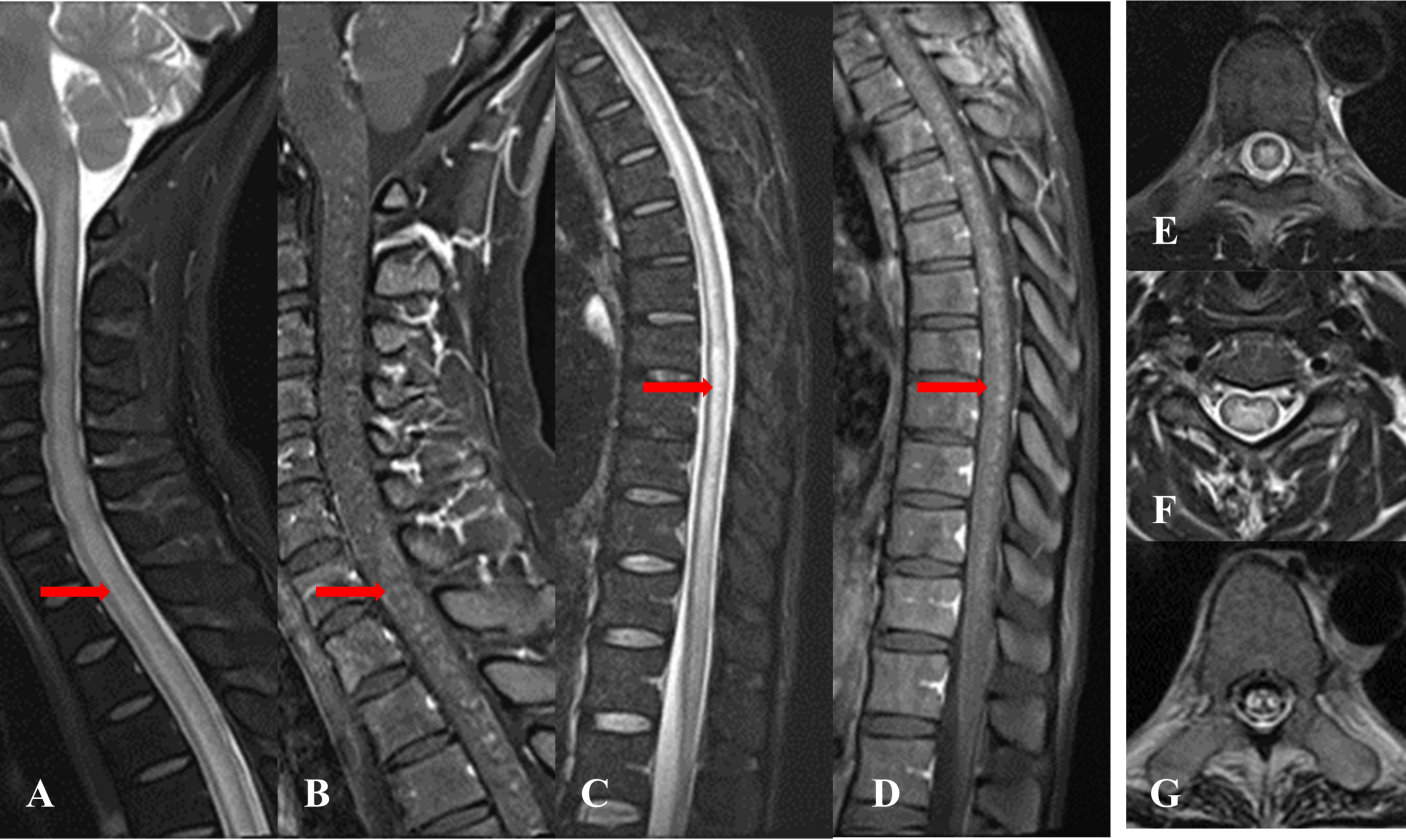
**(A, C, E, F)** Sagittal and axial T2-Short tau with inversion recovery (T2-STIR) of the cervical and thoracic spinal cord in patient #4, with diffuse central gray matter hyperintensity, **(B, D)** Sagittal T1 gadolinium contrast enhanced MRI, of the cervical and thoracic spinal cord, patient #4, with patchy focal enhancement in the cervical and thoracic spinal cord. **(G)** Axial T2-STIR with foci of T2 hyperintensity in the distal thoracic cord, patient #1.

**Figure 3:**
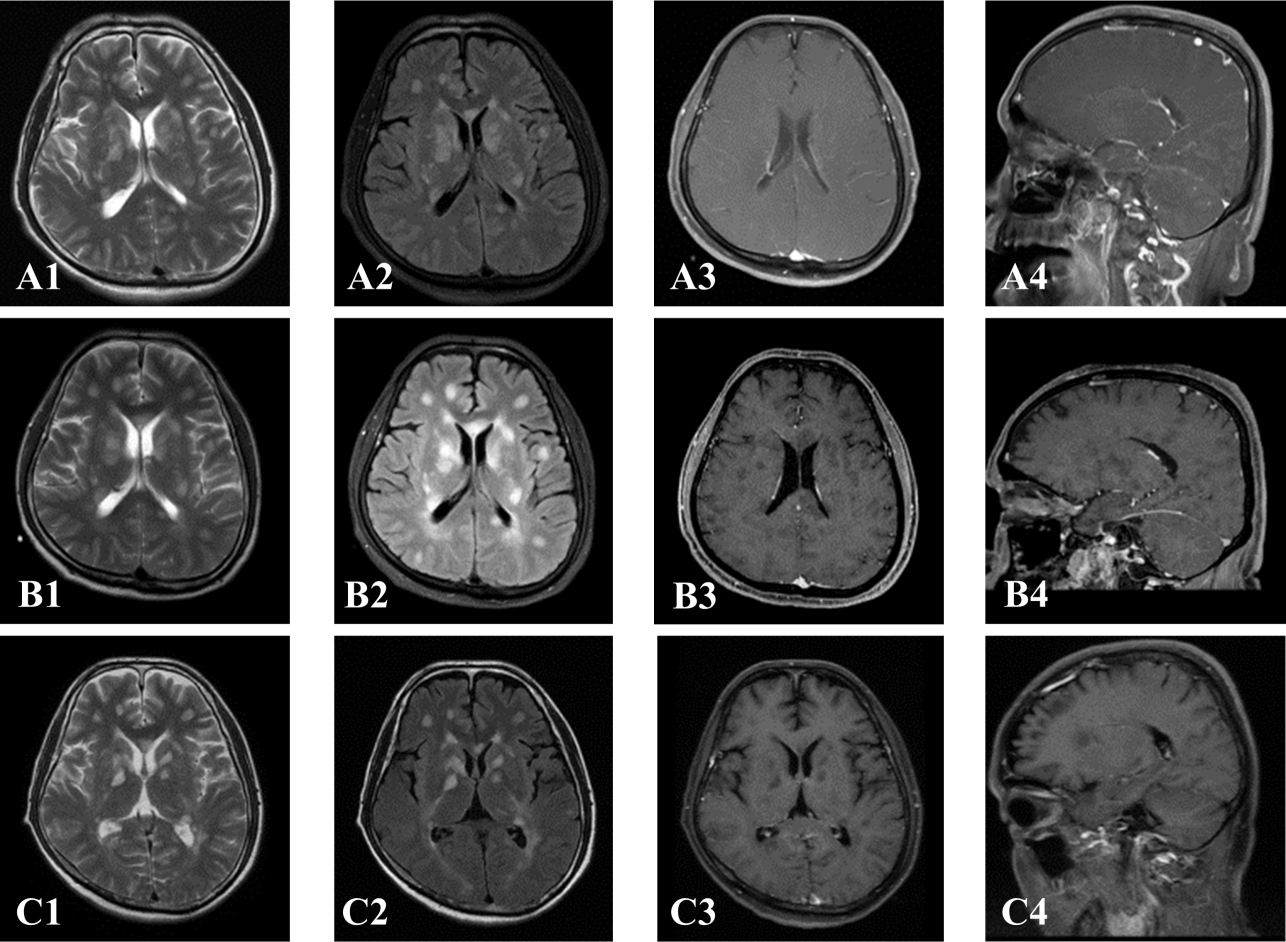
patient #6. Early in the course, multiple patches of hyperintensity in brain **(A1, A2)**, with radial perivascular gadolinium enhancement extending from the lateral ventricles to the white matter (PVRL) **(A3, A4)**, 3 days after treatment, the extent of lesions increased **(B1, B2)**, radial gadolinium enhancement disappeared around the lateral ventricle **(B3, B4)**, 2 weeks after treatment showed that the lesions gradually decreased **(C1, C2)**, radial gadolinium enhancement disappeared **(C3, C4)**.

As described in **Table 3**, MRI of the spinal cord was performed in all 13 patients, and 12 patients had MRI of the brain. For brain MRI, all patients (12 cases) showed multifocal abnormally high signals for T2-weighted (T2WI) and T2-weighted fluid-attenuated inversion recovery (T2-FLAIR) images, showing spots, patches, and lines. T2-FLAIR abnormal high signals were mainly located in the corona radiata, basal ganglia region, brainstem surface, ependyma of fourth ventricle, leptomeninges, centrum semiovale, brainstem, with less than 3 number of lesions located in the corpus callosum, cerebral cortex, hippocampus, and cerebellar hemisphere. Four patients had restricted diffusion weighted images (DWI). For the brain contrast-enhanced T1-weighted MRI (T1WI), three of the 12 patients showed no enhancement, six patients exhibited a radial linear enhancement pattern around the lateral ventricle, five patients presented leptomeningeal enhancement, and one patient showed perivascular patchy enhancement. Notably, three patients who underwent T2-FLAIR gadolinium enhancement after routine scanning, showed more pronounced and extensive leptomeningeal and brainstem surface enhancement compared to contrast-enhanced T1WI (**Figure 1**). Two of the three patients exhibited meningeal enhancement on the surface of the hippocampus. In addition, two of these patients (#1 and #13) showed diffuse abnormal T2W1 hyper-intensity in the corpus callosum with restricted DWI (**Figure 1**). No significant micro-bleeds or iron deposition was observed in patients who underwent craniocerebral SWI examination. Spinal cord MRI revealed T2 hyper-intensity in n= 7 cases with cervical and thoracic spinal cord involvement and n=1 with conus medullaris involvement. One patient (#12) showed multiple short segments, less than three consecutive vertebrae abnormal T2 hyperintensity in the thoracic spinal cord, with six number of patients demonstrating long segments, greater than three consecutive vertebrae of abnormal T2 hyperintensity in the spinal cord central gray matter. The central spinal cord of patient #1 showed obvious “X” or “H” -like T2 hyper-intensity. The lesion in patient #4 involved up to 19 spinal segments, with contrast-enhancement showing extensive spot-like enhancement, accompanied by spinal edema and pia enhancement (**Figure 2**). After treatment with corticosteroids or/and intravenous immunoglobulin, and within 1-5 weeks of the initial pretreatment MRI scan, 10 patients had following up MRIs. In n=4 MRI revealed an increased number of enhancing intraparenchymal lesions and decreased leptomeningeal enhancement compared to the pretreatment MRI. (**Figure 3**) One patient (#1) exhibited a greater abnormal T2 hyperintensity in the thoracic spinal cord compared to prior, while five patients had decreased T2 hyperintensity.

**Table 3:** Distribution of cranial and spinal cord lesions.

Part and examination	Patients, number (%)
Brain MRI	12
abnormal hyperintensity lesions on T2WI-FLAIR	12
Corona radiata	11(91.7)
Basal ganglia region	11(91.7)
Brainstem surface	8(66.7)
Ependyma of fourth ventricle	7(58.3)
Leptomeninges	5(41.7)
Centrum semioval	4(33.3)
Brainstem	4(33.3)
Corpus callosum	3(25.0)
Cerebral cortex	2(16.7)
Hippocampus	2(16.7)
Cerebellar hemisphere	1(8.3)
T1WI gadolinium enhanced lesion (Brain)	12
PVRL	6(50.0)
Leptomeningeal enhancement	5(41.7)
No enhancement	3(25.0)
Patchy enhancement No enhancement	2(16.7)
T2-FLAIR gadolinium enhanced lesion (Brain)	3
Leptomeningeal enhancement	3(100)
Spinal cord MRI	
Cervical cord	7/13(53.4)
Thoracic cord	7/12(58.3)
Lumbar cord	0/7(0)
Long segments	8/9(88.9)
Short segments	1/9(11.1)
T1WI gadolinium enhanced lesion (Spinal cord)	11
Patchy enhancement	5 (45.4)
Leptomendine enhancement	1(9.1)
No enhancement	5(45.4)

In addition to brain and spinal cord lesions, MRI in n=2 patients demonstrated bilateral optic nerve swelling, and abnormal enhancement. Only one patient (#4) presented with clinical symptoms, including blurred vision and decreased vision in both eyes.

### 3.4 Treatment and prognosis

All patients were administered intravenous methylprednisolone during hospitalization, with six patients also receiving concurrent intravenous immunoglobulin. Following treatment, all patients experienced symptom remission and reduced mRS Scores upon discharge (**Table 1**). Anti-GFAP antibody titers decreased or were negative for four patients upon reexamination. All patients continued to use corticosteroids after discharge. One patient had three recurrences and one patient had one recurrence, the latter had a documented history of non-compliance.

## 4 Discussion

In this study there was 8 males (62%) and 4 females (31%) with 1 pediatric patient (7%), with a median age of 33 years +/-38 years, with a broader range of ages of disease onset compared to prior reported studies (8). The onset of GFAP-A exhibited no gender discrepancy, and more commonly manifested in young and middle-aged individuals (8), which may be attributed to the limited number of cases in the study cohort. The predominant clinical manifestations of these 13 patients included limb weakness/numbness, fever, nausea/vomiting, dizziness/headache, as well as bowel and urination dysfunction, consistent with findings from previous studies (9). Most symptoms were non-specific and in four of patients symptoms were accompanied by seizures, tremor, memory loss, visual changes, weight loss, visual and auditory hallucinations. It is worth noting that patients with overlapping antibodies had varying degrees of bladder dysfunction and psychiatric symptoms. These clinical features may be the result of the interaction of different neural antibodies, resulting in clinical manifestations distinct from those of a single antibody. For example, in patients with anti-NMDAR antibodies and demyelinating disease there is an increased likelihood of psychiatric symptoms (10). The coexistence of multiple anti-neuronal antibodies in patients with autoimmune encephalitis may lead to be diverse in clinical manifestations (11). Based on early patient clinical symptomatology it is essential to detect the antibody involved in the early stages of disease. One patient (#12) in our cohort exhibited symptoms consistent with both Alzheimer’s(AD) and Parkinson’s disease (PD), including decreased cognitive function, comprehension, memory, and limb tremor, without a definitive clinical diagnosis. Three patients presented with symptoms of memory impairment. Studies have shown that high anti-GFAP antibody titers are observed in the serum of patients with AD, although the antibody source is not clear (12, 13). Certain researchers feel that serum anti-GFAP antibody cannot be used as a marker for AD, and that antibody titer does not hold diagnostic significance for the condition. In this situation antibodies are only secondary to the destruction of the blood-brain barrier, in which CNS antigens interact with immune cells (14). Research has demonstrated that CSF GFAP can predict AD-related pathological changes and cognitive decline in new-onset PD patients (15). Therefore, further research is needed to investigate the mechanism of action of anti-GFAP antibody and the potential relationship with AD and aging.

In our study, 12 patients presented with prodromal symptoms of fever. Herpes simplex virus type 1 and 2, Epstein-Barr virus, and cytomegalovirus were predominantly detected in the serum, while Epstein-Barr virus was predominant in the CSF. Anti-N-methyl-D-aspartate receptor (NMDAR) encephalitis secondary to herpes simplex virus infection is the most common viral encephalitis. It has been reported that GFAP-A, secondary to herpes simplex virus encephalitis (VE), is characterized by “bimodal encephalitis” (16). There are also case reports of Epstein-Barr virus encephalitis overlapping with GFAP-A (17). In addition, infection is suspected to trigger or enhance the immune response in GFAP astrocytoma (18). Currently, the mechanism by which a viral infection may lead to autoimmune encephalitis (AE) remains unexplained. One possible mechanism is the molecular mimicry hypothesis (19), in which epitopes of pathogen proteins are structurally similar to host proteins, and the antibodies produced against pathogens are cross-reactive with the host, acting as autoantibodies. Additional mechanisms include release of antigens from damaged brain tissue, resulting in the production of anti-synaptic antibodies, triggering a pathological autoimmune response (20). GFAP is an intracellular protein, and we hypothesize that it may be associated with astrocyte exposure to antigens resulting from pathogen infection, potentially leading to autoimmune reactions. Therefore, patients with poor antiviral immunity should undergo testing of the CNS to determine whether complications are due to autoimmune encephalitis. Furthermore, because of the underlying immunologic mechanism, the integrity of the blood-brain barrier is compromised, allowing peripheral antibodies to enter the CNS, leading to the development of oligoclonal bands. Of the five patients in our study who were tested for oligoclonal bands, only four were positive, which could be explained by the time-dependent development of oligoclonal bands, occurs late in the disease course (21).

In our cohort, four patients individually had anti-AQP4 antibodies, anti-MOG antibodies, anti-GM3 antibodies, and anti-NMADR antibodies at the time of admission, these antibodies known as overlapping antibodies. This phenomenon is not uncommon, and studies have shown that anti-NMDAR antibodies are the most prevalent coexisting antibody, followed by anti-AQP4 antibody. When these two antibodies coexist, the presence of ovarian teratoma can be predicted up to 71% of the time (22, 23). The Mayo Clinic (1) found that 38% of patients were diagnosed with a tumor within three years of neurological onset. No tumors were found in our patients as judged by CT of the chest and abdomen and US imaging upon admission, which may be due to small sample size and short follow-up time. In this cohort, there were 10 patients who underwent tumor marker screening with 7 patients showing increases in Neuron-Specific enolase, carbohydrate antigen, ferritin and Non-small cell lung cancer associated antigen. Therefore, whether the patients in this cohort are accompanied by tumors requires long-term dynamic follow-up, and as such corresponding cancer screening of GFAP-A patients should be considered.

The presence of ganglioside antibodies (anti-GM3 antibodies) in patients with GFAP-A has only been reported for one case, known as Brown-Sequared syndrome (24). The specific pathogenic mechanism remains unclear. Ganglioside GM3/GD2 knockout mice exhibit pathological features of reduced myelination, axonal degeneration, and peripheral nerve demyelination, confirming the theory that they are endogenous ligands for myelin-associated glycoprotein (MAG) (25). In adult rats, MAG is enriched in the periaxonal membrane between myelin nodes (26), with anti-GM3 antibodies damaging brain endothelial cells and the blood-brain barrier (27). Therefore, patient (1#) may also have immune damage to the blood-brain barrier and the myelin sheath of the nervous system, caused by anti-GM3.

The latest study (28) of the pathological mechanism of anti-GFAP antibody has found that there are two phenotypes, predominantly lymphocytic and granulomatous, in GFAP-A patients. Despite the absence of astrocyte damage, there is evidence of a cytotoxic T cell-mediated immune response, with an accumulation of CD8+/perforin+/granzyme A/B+ cells surrounding astrocytes with deposition of complement C4d on the astrocytes. This finding further implies that anti-GFAP antibodies themselves do not exert a pathogenic role, and rather implies an inflammatory reaction in CNS, although the origin of anti-GFAP antibodies remains unclear.

We observed pathological changes in the CSF, characterized by elevated white-cell count and protein levels, as well as reduced chloride levels, without a significant decrease in CSF glucose levels. These results are similar to those reported in a previous study (6) and may be related to infection of CNS. Hyponatremia was the predominant electrolyte disturbance observed during hospitalization, consistent with findings of the Kimura study (29) and possibly associated with dysfunction of antidiuretic hormone regulation due to involvement of the supra-optic and paraventricular nuclei of the hypothalamus.

In a previous study (1), 53% of patients exhibited periventricular radial linear (PVRL) enhancement, 33% showed leptomeningeal enhancement, and 71% displayed extensive longitudinal T2 hyperintensity as seen on by spinal MRI. PVRL enhancement and extensive longitudinal T2 hyperintensity in the spinal cord are considered to be typical MRI findings of this disease (30), with one patient having as much PVRL as leptomeningeal enhancement. In this patient the biopsy revealed perivascular inflammatory cell infiltration (CD20 and CD138+) and abundant CD138+ plasma cells in the Virchow-Robin space, without evidence of vascular wall damage (31). The linear enhancement surrounding the ventricle closely corresponded to the perivascular space. In addition, GFAP has a variety of subtypes, GFAPα is the main subtype of the CNS, and the astrocytes expressing GFAP mainly exist in the white matter perivascular regions, while the cerebral cortex and spinal cord are the most abundant gray matter regions. Of note, GFAPδ/ϵ is mainly present in the meninges and sub-ependymal of adults, with high steroid reactivity (32, 33), microglial activation in the meninges, sub-ependymal, and periventricular regions (34). This histopathology seen in GFAP-A is consistent with the imaging manifestations with involvement of the white matter, meninges, periventricular, and ependymal areas. In this study, we believe that the changes in the expression of different subtypes may be the reason for the diverse MRI findings in patients with GFAP astrocytosis, indicating changes in the inflammatory response around intracranial vessels. As such, this may also account for the rapid regression of leptomeningeal and ependymal abnormal signals in our patients after treatment, suggesting that the suppression of GFAPδ/ϵ expression is predominantly involved in this process. Although most of the patients (9/10) showed decreased parenchymal lesions and meningeal enhancement after treatment, four patients showed increased parenchymal lesions despite decreased pia enhancement. Another patient had increased signal intensity in thoracic intramedullary lesions with relief of clinical symptoms, similar to a previous study (32), suggesting that the early stage of GFAP-A is characterized by meningeal lesions, while abnormal hyper-intensities in the brain parenchyma and spinal cord occur later in the disease course, a phenomenon previously reported (31, 35). It is reasonable to believe that there is a “delay” phenomenon between the clinical manifestations and imaging findings are not fully synchronized. There existed a temporal dependence between imaging findings and clinical manifestations within 1-5 weeks after initial treatment. Continuouscontrast-enhanced MRI can help to more comprehensively understand disease course and enable early diagnosis of the disease. Patients at risk for GFAP-A should have continuous monitoring of the CNS and spinal cord via MRI. The PVRL pattern enhancement is not exclusive to GFAP-A, a similar enhancement patterns is observed anti-GFAP antibody negative patients with lymphoma, leading researchers to propose this as “GFAP-A-like” pattern of enhancement (30, 36). A key differentiating factor is GFAP-IgG, and the variable leptomeningeal enhancement reported in studies. In this study, there was a higher prevalence of leptomeningeal enhancement and brainstem surface enhancement compared to previous reports (1, 6). In this cohort, the majority of patients (8/13) demonstrated with T2-FLAIR hyperintensity along the surface of the brainstem. Three patients had T2-FLAIR gadolinium-enhanced scanning, with more pronounced and extensive T2 hyperintensity along the brain stem surface and leptomeninges than T1 weighted post-gadolinium sequences. This is because T2-FLAIR, with its high sensitivity to fluid, can provide more information than MPRAGE imaging. T2-FLAIR gadolinium-enhanced scanning can only show enhancement along the leptomeninges with blood brain barrier breakdown, not vascular enhancement. Utilization of T2-FLAIR pre and post-gadolinium sequences and subtraction techniques can reduce the number of false negative image findings on MRI (37, 38). Therefore, in routine scanning sequences, the abnormally high signal intensity of T2-FLAIR along the surface of the brain stem should be taken seriously. In addition to pathological conditions such as bacterial meningitis, T2-FLAIR sequences with gadolinium may provide clues such as leptomeningeal enhancement and aid in an earlier diagnosis of suspected autoimmune encephalitis. This approach can improve the detection rate of leptomeningeal lesions and facilitate early diagnosis, treatment, and prognosis.

Three patients in the study cohort had corpus callosum involvement presenting as reversible splenial lesion syndrome (RESLES). As previously reported (39, 40), our patient also had prodromal symptoms and viral infection. However, unlike the previous study reporting only oval lesions in the splenial corpus callosum (SCC), two patients presented with diffuse abnormal T2 hyperintensity and ADC restricted diffusion with DWI hyperintensity. RESLES are generally classified into two types (41): type I, characterized by isolated oval lesion in central corpus callosum, and type II, characterized by a lesion that extends throughout the corpus callosum and into adjacent white matter regions. RESLES is typically secondary to viral encephalitis/encephalopathy, and its clinical manifestations are nonspecific. Autoimmune GFAP astrocytopathy with RESLES is still very rare and its pathological basis is still unclear. Cytokine-mediated cytotoxic edema (41), possibly related to viral infection or coexisting autoimmunity (39, 42), has been speculated as a possible cause. In this cohort, patients presented with the less common type II RESLES, possibly a pathological change secondary to GFAP astrocytoma and associated viral infection.

The pathogenic mechanism by which autoimmune GFAP-A results in changes in the optic nerve is unclear. *In vivo* tracer experiments in mice and results from human autopsy have confirmed that CSF can enter the optic nerve through the perivascular space (43). The loss of retinal ganglion cells due to optic neuritis is mediated by astrocyte complement C3 (44). Therefore, we hypothesize that the immune cells in the patient’s CSF may interact with the optic glial cells in this manner, potentially leading to damage of the optic nerve and retina. Research has indicated that anti-GFAP antibody also exhibits cross-reactivity with endoplasmic reticulum resident protein 57 (ERP57) on the cell membrane, resulting in alterations in cell signaling and ultimately providing cellular protection against oxidative stress. In an experimental autoimmune encephalomyelitis model, astrocyte yes-associated protein (YAP) up-regulates TGF-β signaling to safeguard the optic nerve and retina (7, 45). This may account for the abnormal image findings for the bilateral optic nerves of one of the patients, who did not have corresponding clinical manifestations. It is possible that autoimmune injury triggers concurrent activation of protective mechanisms, preventing involvement of retinal cells. In addition, one patient (3#), despite clinical presentation with blurred vision, had no image abnormalities of the optic nerves and had high titers of anti-MOG antibodies (1:1000) in serum, MOG-related diseases (MOGAD) are mainly associated with adult optic neuritis (46).

Consistent with previous research (34), spinal cord MRI demonstrated involvement of the central gray matter and a higher prevalence of long segments, potentially correlating with clinical symptoms such as limb weakness, numbness, and bladder dysfunction. Conus medullaris was not involved in our patients, but studies have reported (31) that the contrast enhancement in the leptomeninges surrounding the conus can occasionally be observed. With the exception of one patient mentioned earlier (1#), both patchy enhancement of the spinal cord and enhancement of the leptomeninges on the surface of the spinal cord were reduced after steroid treatment. Neuromyelitis optica spectrum disorders (NMOSD) presents with long segment lesions. The “H” sign is more commonly observed in MOGAD myelitis (47). Therefore, the detection of anti-GFAP antibody in serum or CSF is crucial for the diagnosis of GFAP-A.

In our cohort, all patients responded well to immunotherapy, clinical symptoms improved at discharge, and CSF antibody titers decreased or turned negative upon reexamination. This finding further supports the notion (22, 48) that fluctuations in antibody titer are correlated with disease severity and prognosis, suggesting that CSF anti-GFAP antibodies may serve as a biomarker reflecting the efficacy of acute phase treatment. Further, recurrent MOGAD is prone to relapse after tapering of steroids and discontinuation of treatment (49). GFAP-A is also a steroid-dependent autoimmune disease, so relapsing patients may be due to self-withdrawal or rapid reduction. Patients should continue steroid use and undergo gradual tapering after discharge. Continuous detection of antibody titers in serum or CSF of patients is essential for disease surveillance and to facilitate the development of appropriate treatment strategies, thereby preventing recurrence and improving prognosis.

## 5 Conclusion

In this study there were slightly more male patients than female patients. Encephalitis and encephalomyelitis were the most common clinical phenotypes, often accompanied by hyponatremia and hypoglycemia. Most patients presented with unexplained fever symptoms and viral infections prior to acute exacerbation. Overlapping antibodies are common, and we found anti-GM3 antibodies, a rare finding also previously reported, although the specific mechanism of induction is unclear. The number of cells and protein levels in CSF were elevated, and oligoclonal bands were detected in some cases. While certain patients exhibited increased tumor markers, no definitive tumor was identified. Anti-GFAP antibody may not be the causative agent of the disease and the origin of the antibody is unknown. However cytotoxic T cells are believed to play a significant role. Cerebral MRI commonly identified involvement of the corona radiata, basal ganglia region, brainstem surface, ependyma of fourth ventricle, leptomeninges, centrum semiovale, brainstem. The optic nerve and corpus callosum were rarely involved. Periventricular linear radial enhancement and leptomeningeal enhancement were typical patterns, especially when the T2-FLAIR gadolinium enhanced scan was performed. Most of the spinal cord MRIs showed extensive longitudinal T2 hyper-intensity, centrally distributed and involving the gray matter, with patchy and pial enhancement. Bilateral optic nerves were also involved. In addition to the classic reversible splenium of corpus callosum syndrome (type I), we also found the much rarer type II RESLES. Patients may exhibit sensitivity to steroid therapy, with relief of clinical symptoms and improved image findings following treatment. However, a “delayed” phenomenon can occur in some cases. There existed a temporal dependence between imaging findings and clinical manifestations. Therefore, aside from the typical enhancement pattern of PVRL, we should be attentive to the abnormally hyperintensity of T2-FLAIR along the surface of the brain stem, which is also of indicative significance for the early diagnosis of this disease. Simultaneously, early T2-FLAIR gadolinium enhanced scan or even the subtraction technique are valuable for the detection of early lesions and accurate diagnosis. The majority of patients had a favorable prognosis and only a few experienced relapse.

## 6 Limitations

First, all data were retrospectively obtained from electronic medical records, and only patients who were still in the same hospital after relapse were identified as relapsing patients. Second, the sample size is small, and future studies should be conducted with a larger population. It is important to note that variations in scanning equipment and sequences across different hospitals may impact disease site identification and patterns of intensification.

## Data Availability

The original contributions presented in the study are included in the article/**Supplementary Material**. Further inquiries can be directed to the corresponding author.
